# Rectal Carcinoma Case Causing Bicytopenia

**DOI:** 10.14740/wjon784w

**Published:** 2014-03-11

**Authors:** Sami Cifci, Aynur Ugur Bilgin, Murat Biyik, Huseyin Ataseven, Hatice Toy

**Affiliations:** a Department of Gastroenterology, Konya NEU Meram Faculty of Medicine, Turkey; b Department of Hematology, Konya NEU Meram Faculty of Medicine, Turkey; c Department of Pathology, Konya NEU Meram Faculty of Medicine, Turkey

**Keywords:** Bicytopenia, Rectal carsinoma

## Abstract

Although conditions leading to bicytopenia and pancytopenia secondary to infiltrative diseases of the bone marrow are seen, a profound anemia or hemorrhages are frequently observed in such cases. As bone marrow infiltrations may be associated with primary hematological diseases such as leukemia, lymphoma or myeloma, rarely they may also be associated with solid tumor metastases. Here we have presented a case of rectal carcinoma causing profound bicytopenia dependent on diffuse bone marrow involvement.

## Introduction

Bone marrow infiltrations among solid tumors are mostly seen in breast, prostate, lung and thyroid carcinomas [1]. Rectum cancers spread lymphatically, hematogenously, adjacently and transperitoneally. The most frequently observed metastasis regions are regional lymph nodes, liver, lung and peritoneum. Approximately 20% of the patients diagnosed with colorectal cancer have already developed distant metastasis at the time of diagnosis [2].

Venous drainage of the rectal region is via superior, middle and inferior hemorrhoidal veins. While superior hemorrhoidal vein links to portal system by draining into inferior mesenteric vein, middle and inferior hemorrhoidal veins link to systemic circulation by inferior vena cava. Therefore, while in distal rectal tumors initially distant metastasis can be observed in tissues such as lung, bone, at other regions, the most frequent metastasis is in liver followed by tissues such as lung and bone. Moreover, pelvic and lumbosacral vertebral metastasis may be observed as a result of hematogenous spread via Batson vertebral venous plexus [3]. Symptomatic bone metastasis is not very common in rectal carcinomas in a trial examining a wide series of cases in patients with rectal carcinoma, it has been determined that the ratio of bone metastasis is below 10% [4].

Here we have presented a case of rectal carcinoma causing profound bicytopenia dependent on diffuse bone marrow involvement.

## Case Report

**Figure 1:**
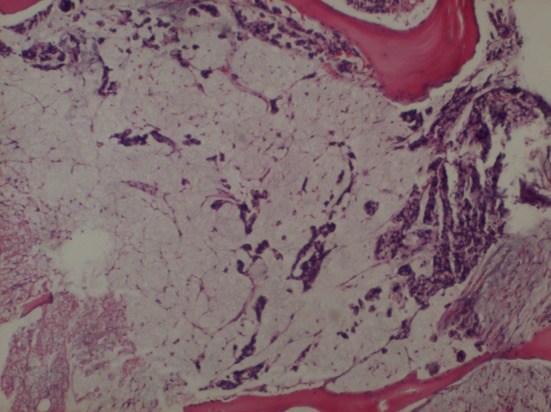
Bone morrow biopsy examination. There were atypic looking mucin islets and tumor cell groups occupying bone marrow spaces (× 4).

**Figure 2:**
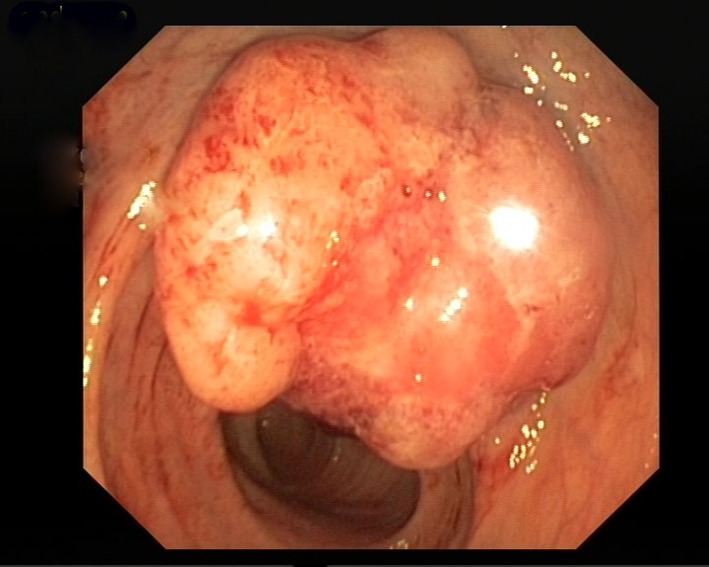
Colonoscopy examination. Performed thereupon a massive lesion has been detected at rectum.

A 60-year-old female patient, with a history of cholecystectomy approximately 1 month ago and a history of using high-dose analgesics due to low back pain, has applied to the emergency service of our hospital with the complaint of rectal bleeding lasting for a week and due to general condition disorder. It has been indicated that routine tests performed at the control visit of the patient approximately 2 weeks ago were normal. In the initial tests performed at the emergency service, the following results were found: WBC: 8.6 × 10^9^/L, Hb: 8.52 g/dL, PLT: 22 × 10^9^/L, AST: 64 U/L, ALT: 46 U/L, LDH: 850 U/L, T. bil: 1.88 mg/dL and D. Bil: 0.79 mg/dL. Patient has been admitted to the hematology clinic with the pre-diagnosis of acute leukemia. In peripheral smear examination, no atypical cell has been observed. In abdominal ultrasonography, no pathology has been observed. Bone marrow aspiration biopsy performed has resulted as dry tap. In bone marrow imprint examination, it has been determined that cells with atypical nucleoli were compliant with distant organ metastasis causing syncytium. In bone morrow biopsy examination, there were atypic looking mucin islets and tumor cell groups occupying bone marrow spaces, among calcific bone spicules (Fig. 1). No pathology has been observed in upper endoscopic examination; in colonoscopy examination performed thereupon a massive lesion of 14 to 18 cm has been detected at rectum (Fig. 2). Mass biopsy has been observed to be consistent with rectal carcinoma. Patient, considered to have bone marrow infiltration of rectal carcinoma, has been referred to the oncology clinic for the organization of her treatment.

## Discussion

In an old patient applying with hemorrhage findings and bicytopenia, a manifestation of acute leucosis has been considered firstly; a rectal carcinoma case, which may cause thrombocytopenia to this extent, is not common. Although symptomatic bone metastasis has been observed in colorectal region tumors relatively more, consideration of bone marrow metastasis has not been widely accepted to this extent. When literature review is performed, it will be found that very limited information is available on this subject, and that diffuse bone marrow involvement is very rarely seen.

Although examination of bone marrow involvement in colorectal cancers is not routinely used, in fact, there are studies supporting that spread of the disease at micro-metastasis level is not all that rare. In a study published in 2010, bone marrow metastasis has been examined by using reverse transcription polymerase chain reaction (RT-PCR) and immuno-histochemical staining methods with cytokeratin, and when independent malign invasion rates at 20% and 33% and survival rates have been compared, it has been determined that survival was significantly longer in RT-PCR-negative patients [5]. And in another trial on approximately 245 patients with colorectal tumor, bone marrow has been examined with cytokeratin stain, and positive metastatic cells have been seen in bone marrow of 12% of patients [6].

With the development of new techniques that are able to demonstrate bone marrow involvement, beyond the classical classifications used for colorectal cancers today, establishment of new classifications and development of new treatment modalities seem to be unavoidable.
